# Progress and Research Trends on *Catha edulis* (Vahl) Endl. (*Catha edulis*): A Review and Bibliometric Analysis

**DOI:** 10.3389/fphar.2021.705376

**Published:** 2021-11-11

**Authors:** Shuang Ye, Jin Hu, Zilong Liu, Man Liang

**Affiliations:** ^1^ Department of Forensic Medicine, Tongji Medical College, Huazhong University of Science and Technology, Wuhan, China; ^2^ Department of Otolaryngology-Head and Neck Surgery, Tongji Hospital, Tongji Medical College, Huazhong University of Science and Technology, Wuhan, China

**Keywords:** bibliometric analysis, cathinone, chemical composition, toxicology, pharmacological effect, mechanisms, C*atha edulis* (vahl) endl., *Catha edulis*

## Abstract

*Catha edulis* (Vahl) Endl., known as *Catha edulis* or Khat is a traditional and regional plant for chewing, smoking and drinking, that has posed a worldwide public health problem due to its recent emerging abused consumption. In the face of the massive use of *Catha edulis*, we reviewed related publications to analyze the progress and research trends through bibliometric methods. After screening, a total of 514 scientific publications published from 1997 to 2020 were included by systematic retrieval from the Web of Science (WoS) database. According to further scientometric analysis, the annual number of publications output kept rising in most of the years. Ethiopia and the United States of America (USA) have been devoting significant contributions to the field. Though the research emphasis had been the chemical composition and pharmacological and toxicological effects for several years, the hot spots were transferred; the mechanism investigations of *Catha edulis* have been the focus in recent years, which might be continued in the future. Furthermore, co-operations of multi-disciplinary researchers are needed to minimize abuse harms and maximize the medicinal benefits of *Catha edulis* to human beings.

## Introduction

*Catha edulis* (Vahl) Endl., usually called *Catha edulis* or Khat, is a native plant in parts of East Africa and the Arabian Peninsula, and is commonly chewed as natural stimulants in local practice and habits (Graziani et al., 2008). It is also frequently used in other countries with different names, like “qat” in Yemen, “chat” in Ethiopia, “qaad” or “jaad” in Somalia, and “mirra” in Kenya (Kandari et al., 2014). Initially, *Catha edulis* was traditionally governed in the regions for purpose of religious, ritualistic, and medicinal use for several decades (Gebissa, 2004). In the early 1990s, *Catha edulis* spread to Europe, North America, and Australia due to emigration from the Horn of Africa (Al-Hebshi and Skaug, 2005; Gebissa, 2010). Nowadays, there are estimated to be over 20 million abusers globally, including both male and female adults, and college and middle school students (Riyaz et al., 2014; Odenwald and Al'Absi, 2017).

The main active ingredients of *Catha edulis* include phenylpropylamino alkaloids, cathine, and cathinone, which are responsible for the pharmacological and toxicological effects. Among these, the β-keto analogue of amphetamine from a structural perspective could lead to dependence via psychostimulatory effects on the nervous system (Elmai, 1983; Morghemm and Rufatm, 1983; Kalix, 1987; Giannini et al., 1992; Omar et al., 2015). Additionally, prolonged exposure to *Catha edulis* could lead to dependence, psychosis, hypertension, cardiovascular complications, sexual dysfunction, hepatotoxicity, etc (Odenwald and Al'Absi, 2017). Therefore, *Catha edulis* abuse has become one of the most serious public health concerns. However, except for such adverse outcomes, there are other reports suggesting potential medicinal benefits of *Catha edulis* such as antibacterial activity (Al-Hebshi et al., 2006), antidepressant-like activity (Alfaifi et al., 2017), adjunct treatment of obesity (Hauner et al., 2017), and neural tissue substitutes (Abdel Bary and Harmal, 2019a), which deserve comprehensive analysis.

In this study, we aimed to elucidate the progress and hotspots of *Catha edulis* research via a bibliometric analysis and aggregate the opinions of effects and mechanisms, which would help relieve the relative health concern (Zupic and Cater, 2015). This bibliometric net analysis aims to give an overview of *Catha edulis* research and reveal the directions and frontiers for future development.

## Methods and Materials

**FIGURE 1:**
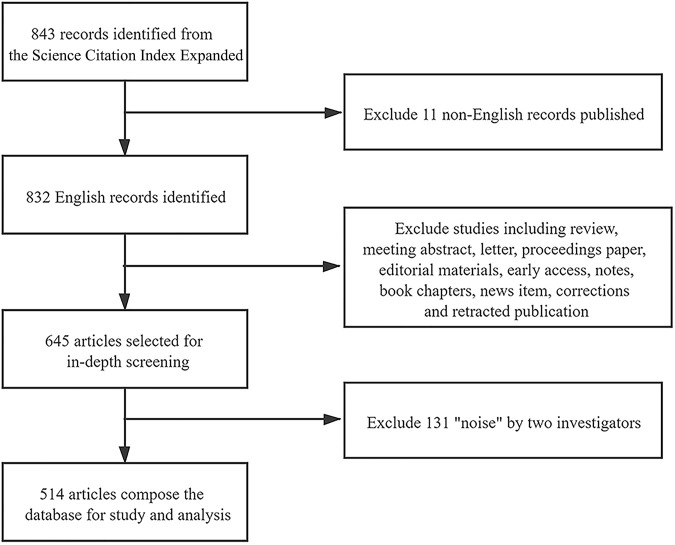
The flow chart of database construction.

The WoS database is the most frequently used for search in various scientific fields and retrieving related literature. And the WoS core collection indexes scientific journals exerting greater impact. For accurate and representative search, we collected index topics of *Catha edulis* from Medical Subject Headings (MeSH) list in PubMed and built an initial database from the WoS core collection with the searching strategy: [(Topic = (“Khat”) OR (“Khats”) OR (“Catha”) OR (“Catha eduli”) OR (“*Catha edulis*”) OR (“edulis, Catha”) OR (“Qat Plant”) OR (“Qat Plants”) OR (“Plant, Qat”) OR (“Plants, Qat”) OR (“Miraa”)]. Though research on *Catha edulis* started in 1952, early publications have proved to be of low impact and output, or published in journals with no official IF or low SJR like local African journals (Gaillard J, 1992). From 1997 to the present, a continuous emergence of research productivity was observed, and critical research performance related to *Catha edulis* has progressed which deserves a scientometric analysis. Based on the retrieval period between 1997 to Dec 21st, 2020, 843 records were identified. Since English served as the language of worldwide scientific communication, we excluded 11 non-English records and narrowed 832 English studies for further analysis. To detect original discoveries, we restricted document types to original articles and obtained 645 science publications. After downloading the raw data of these aforementioned articles, two investigators individually checked and eliminated noise, any divergence was reconciled, and a consensus was finally achieved through discussion. Finally, 514 publications composed the “*Catha edulis*” database (Figure 1). Statistical analyses of the variables of the database were conducted by CiteSpace 5.7.R1 to generate visualized graphs, including distribution of publication outputs, collaborations between countries, and co-occurrence and burst keywords for detection of research trends and frontiers (Chen, 2004; Chen, 2006; Chen et al., 2010).

## Results

### Publication Outputs and Growth Trend

**FIGURE 2:**
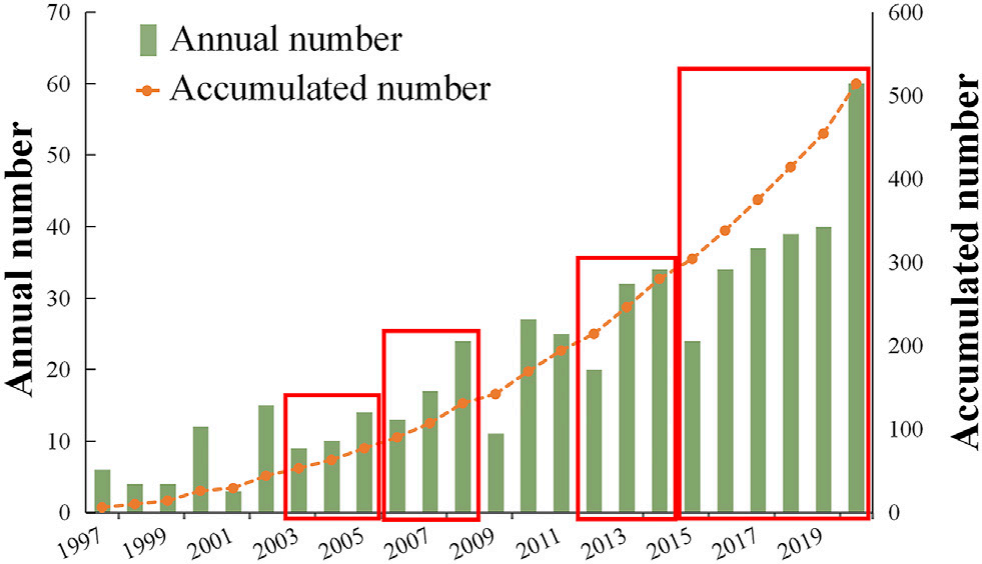
Annual number and accumulated number of *Catha edulis* publications from 1997 to 2020.

The annual publication outputs had a fluctuating growth rate during most times, and the fluctuation was periodic over the past 24 years according to the histogram. The number of annual publications increased dramatically in 2020, by 10-fold from 6 in 1997 to 60 in 2020 (Figure 2). The Mann-Kendall (MK) test was applied to the annual publication number, and the statistics results (Z = 5.407 > 2.32>0) indicated a significant increasing trend of the annual outputs related to *Catha edulis* with a 0.001 level of significance.

### Contributed Countries and Institutions

**FIGURE 3:**
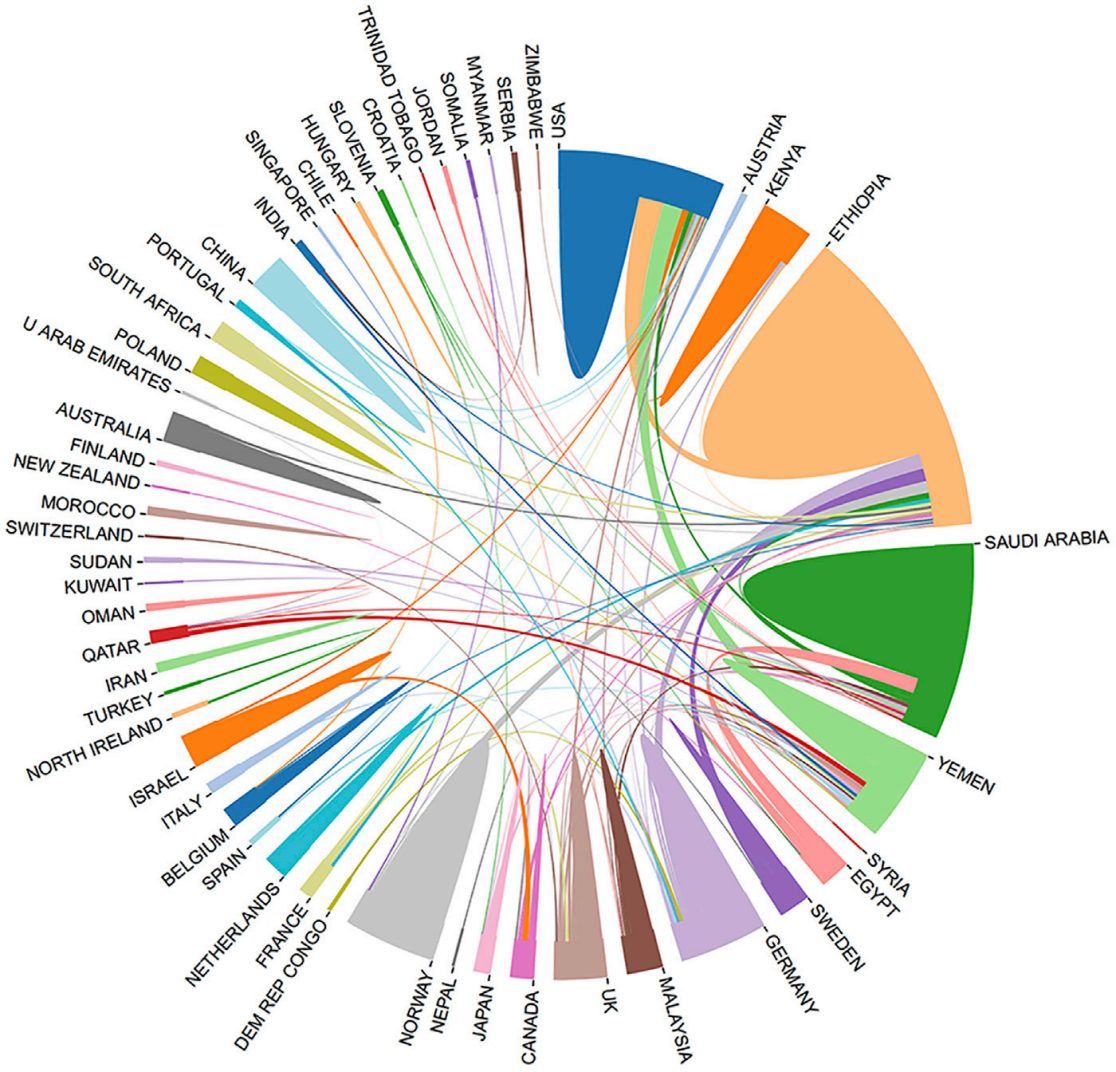
Cooperation between contributed countries.

According to the WoS, the retrieved publications on *Catha edulis* were indicated to be contributed by 65 countries/regions with intensive cooperation. From this sensitive indicator of reflecting attention and strength commanded by a country/region in a specific research area, the most productive countries were Ethiopia (32.9%, 169/514), followed by the USA (16.7%, 86/514), Saudi Arabia (12.6%, 65/514), and Yemen (12.3%, 63/514) (Table 1; Figure 3). Half of the top ten most productive countries were located in East Africa and the Arabian Peninsula, including Ethiopia, Saudi Arabia, Yemen, Egypt, and Kenya, which produced approximately two-thirds (67.7%) of the publications on *Catha edulis* in total. In addition, North American and European countries contributed several research achievements in recent years.

**TABLE 1:** The top 10 most productive countries and institutions for *Catha edulis* publications.

Country or region	Articles (%^a^)	Citations	H-Index	Citations per article	Top Institutions	Articles by top Institutions (%^b^)
Ethiopia	169 (32.9%)	1759	23	10.41	Addis Ababa University	62 (36.7%)
USA	86 (16.7%)	1,200	20	13.95	University of Minnesota System	23 (26.7%)
Saudi Arabia	65 (12.6%)	514	14	7.91	Jazan University	33 (50.8%)
Yemen	63 (12.3%)	1,033	20	16.40	Sana’a University	38 (60.3%)
England	42 (8.2%)	1,410	16	33.57	University of London	21 (50.0%)
Germany	38 (7.4%)	774	13	20.37	Goethe University Frankfurt	6 (15.8%)
Egypt	26 (5.1%)	176	8	6.77	Assiut University	5 (19.2%)
Kenya	25 (4.9%)	337	11	13.48	University of Nairobi	17 (68.0%)
Norway	22 (4.3%)	418	14	19.00	University of Bergen	14 (63.6%)
Malaysia	20 (3.9%)	100	6	5.00	University Malaya	10 (50.0%)
East Africa and Arabian Peninsula	348 (67.7%)	3,819	25	10.97	Addis Ababa University	62 (17.8%)

a Percentage indicates the ratio of the number of articles on *Catha edulis* published in this country or region to that in the “*Catha edulis*” database (514).

b Percentage indicates the ratio of the number of articles on *Catha edulis* published by top institutions to that by their country or region.

The total number of citations for publications in this field was 7978, giving an average citation per article of 15.52 (7978/514). The top three countries of citations per article were England (33.57, 1,410/42), Germany (20.37, 774/38), and Norway (19.00, 418/22). Moreover, the average H-index of the retrieved papers queried on the WoS was 42. Ethiopia with the highest H-index of 23 was the most effervescent, followed by the USA (20) and Yemen (20). Furthermore, the core institutions in the countries, such as Kenya (68.0%), Norway (63.6%), and Yemen (60.3%), completed the majority of achievements.

Figure 3 also shows the cooperation between contributing countries: Ethiopia, as the most collaborative country, cooperating with 32 countries, followed by Saudi Arabia and the USA, maintaining close cooperation with 27 and 20 countries, respectively. These collaborations with other countries and regions played a significant role in *Catha edulis* research.

### Highly Cited Publications

In order to identify influential representative publications, we listed the top 10 most cited articles (Table 2). This high-quality scientific research was all published in authoritative journals categorized in pharmacology (No.1), toxicology (No.2, 7, 9, 10), psychiatry (No.3), analytical chemistry (No.4), and public health (No.5, 6, 8), with Impact Factor (IF, 2020), 5-Year Impact Factor (IF5) and SCImago Journal Rank (SJR) retrieved. In these interdisciplinary journals, the most-cited research involved phyto-research and social issues. Germany, Ethiopia, and Yemen contributed two of the top 10 articles, respectively; and the IF5 and SJR of the top two countries were Germany (IF5: 10.249, SJR: 3.463) and Ethiopia (IF5: 5.706, SJR: 2.849).

**TABLE 2:** The characteristics of highly cited articles.

Rank	Total citations	Article title	Journal	Published year	Country	IF 2020	IF 5-years	SJR 2020
1	147	Pharmacokinetics of cathinone, cathine and norephedrine after the chewing of khat leaves	British Journal of Clinical Pharmacology	2003	Germany	4.335	4.902	1.216
2	128	Clinical characteristics of mephedrone toxicity reported to the UK National Poisons Information Service	Emergency Medicine Journal	2011	England	2.740	3.135	0.708
3	122	Khat use as risk factor for psychotic disorders: A cross-sectional and case-control study in Somalia	BMC Medicine	2005	Germany	8.775	10.249	3.463
4	107	Determination of cathinones and related ephedrines in forensic whole-blood samples by liquid-chromatography-electrospray tandem mass spectrometry	Journal of Chromatography B: Analytical Technologies in the Biomedical and Life Sciences	2011	Denmark	3.205	3.068	0.729
5	87	Khat and alcohol use and risky sex behaviour among in-school and out-of-school youth in Ethiopia	BMC Public Health	2005	Ethiopia	3.295	4.003	1.230
6	87	The prevalence and socio-demographic correlates of khat chewing in Butajira, Ethiopia	Acta Psychiatrica Scandinavica	1999	Ethiopia	6.392	5.706	2.849
7	82	Investigation into the toxicological effects of *Catha edulis* leaves: a short term study in animals	Phytotherapy Research	2002	Yemen	5.878	5.286	1.019
8	79	Khat chewing is a risk factor for acute myocardial infarction: a case-control study	British Journal of Clinical Pharmacology	2005	Wales	4.335	4.902	1.216
9	78	Khat (*Catha edulis*) consumption causes genotoxic effects in humans	International Journal of Cancer	2001	Austria	5.145		
10	75	Toxicological evaluation of *Catha edulis* leaves: a long term feeding experiment in animals	J Ethnopharmacol	2002	Yemen	3.690		

### Hot Spots of Catha Edulis Research

**FIGURE 4:**
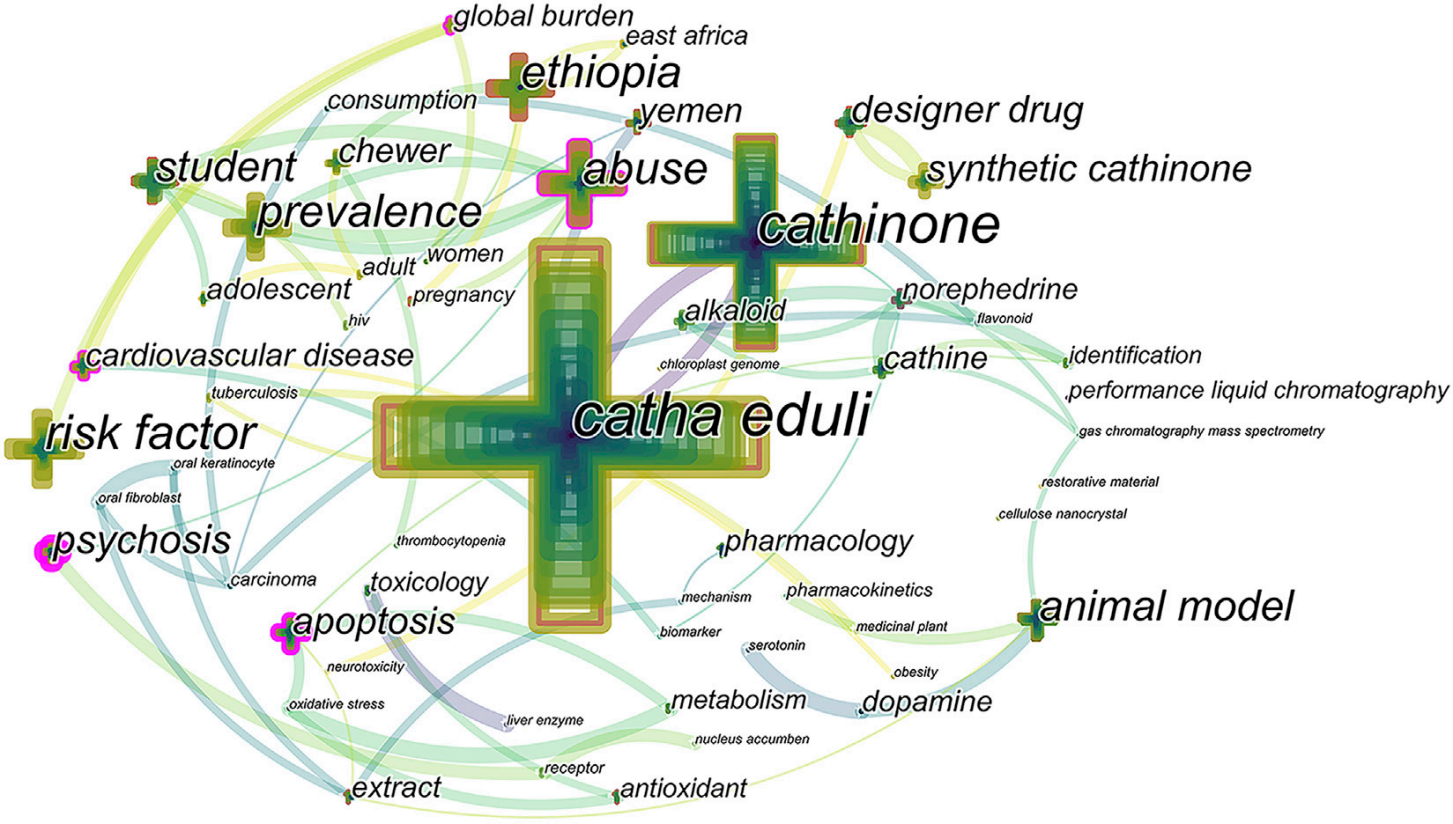
Network of keywords co-occurrence. The cross size and the link each illustrated the frequency and relevance of keywords.

In order to analyze and visualize the network of keyword co-occurrence, we excluded repeated and irrelevant keywords, and prune-sliced and merged the networks using CiteSpace (threshold of occurrences frequency at 5, Figure 4). This map illustrated the frequency and relevance of keywords according to cross size and link. High frequency keywords included “catha eduli” (frequency: 362, centrality: 0.13), “cathinone” (frequency: 141, centrality: 0.12), “risk factor” (frequency: 58, centrality: 0.09), “abuse” (frequency: 57, centrality: 0.34), and “Ethiopia” (frequency: 55, centrality: 0.17), represented by larger crosses. Other keywords such as “prevalence” (frequency: 48, centrality: 0.05), “animal model” (frequency: 42, centrality: 0.20), “student” (frequency: 36, centrality: 0.01), “psychosis” (frequency: 29, centrality: 0.16), and “apoptosis” (frequency: 28, centrality: 0.13) showed a moderate frequency.

### Scientific Landscapes of Research Trends

**FIGURE 5:**
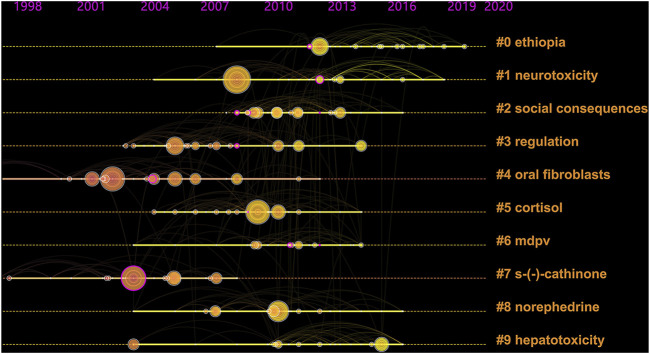
Co-cited references timeline map of *Catha edulis*. Nodes on the map represent referenced documents. The size of nodes represents the frequencies of cited references, and the location reflects the present time. And years are arranged horizontally at the top. The clusters are performed based on the themes of co-cited references and the label of each cluster is shown at the end of the timeline.

The co-cited references were clustered and identified *via* a timeline map (Figure 5), which can help identify development trends of the specific research field (Niazi and Hussain, 2011), indicate the shift of research concerns, and shape the specialty structure of knowledge. According to the timeline map of co-cited references, 10 clusters were analyzed in depth, most of which were concentrated in the period from 2003 to 2016. The earlier studies were mainly devoted to oral fibroblasts (#4) and s-(-)-cathinone (#7). Then the research focus shifted to regulation (#3), neurotoxicity (#1), cortisol (#5), norephedrine (#8), social consequences (#2), Ethiopia (#0), and hepatotoxicity (#9) which were involved in chemical composition, toxicology and global prevalence of *Catha edulis*. MDPV (#6), as one of the synthetic cathinones with a similar structure of cathinone, a main ingredient of *Catha edulis*, was focused on as well.

**FIGURE 6:**
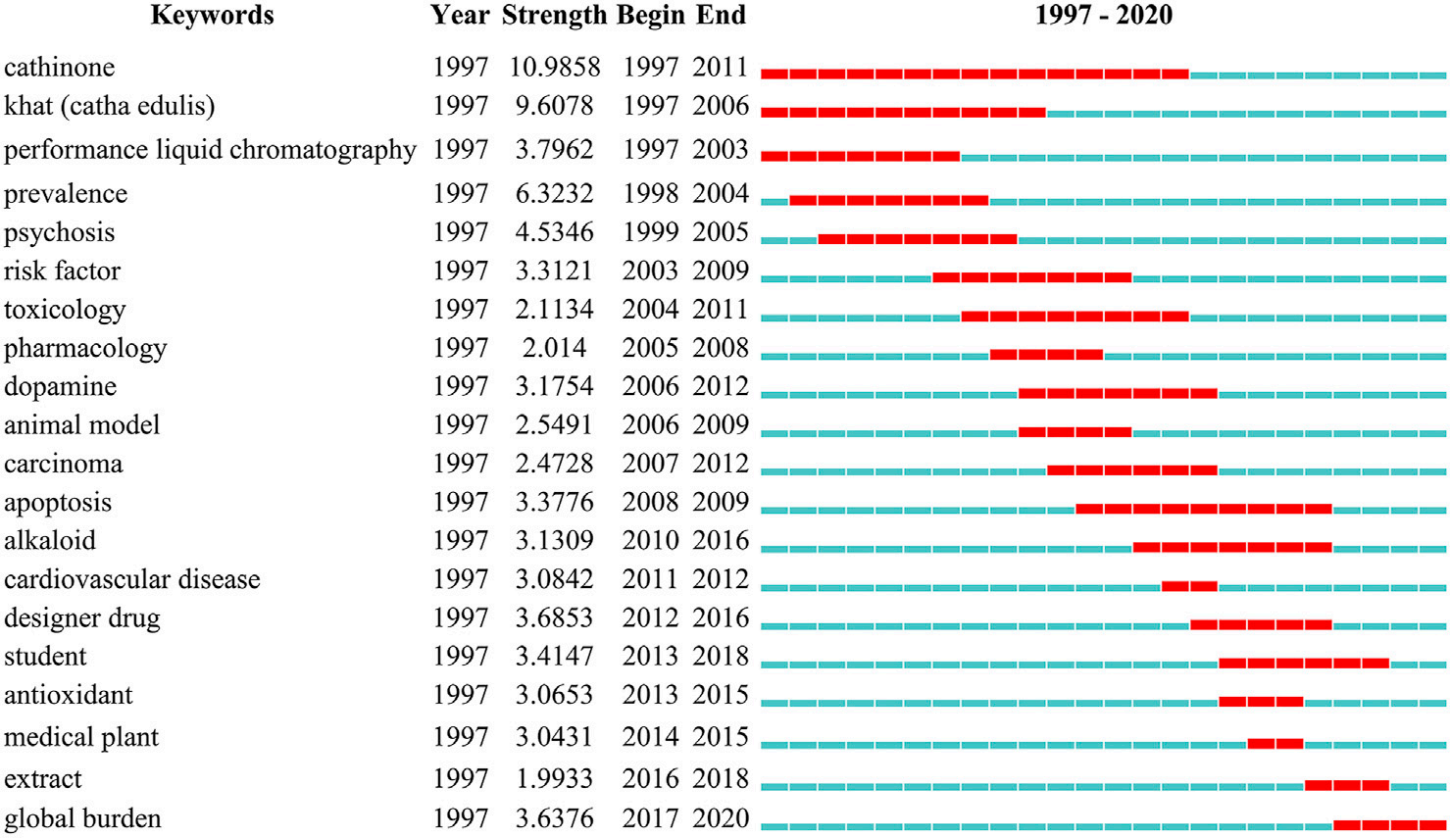
Top 20 keywords with the strongest citation bursts. Each blue or red short line represents a year, and a red line stands for a burst detected year.

Keywords could summarize the subject and content of the publication, which play an essential role in tracing hotspot transfers in scientific research. A keyword burst map was obtained for visualization of keywords with the strongest bursts in scientific articles by CiteSpace algorithm-dependent analytical tool and summarization of focus transfer according to their time of appearance (Figure 6). Besides, burst keywords can indicate prominent research topics studied over different periods by reflecting the intensity and duration of hot spot issues, while the latest burst keywords represent up-to-date transfers of research focus (Kleinberg, 2003; Chen et al., 2014). Based on the top 20 keywords with the strongest citation bursts, the early research focus was to explore the chemical composition of *Catha edulis* (cathinone), identification (high-performance liquid chromatography), and prevalence. Then, the burst keywords were transferred to 1) health harms (psychosis, carcinoma, cardiovascular disease, and risk factor), 2) toxicology (apoptosis) and pharmacology (alkaloid), and 3) mechanism (antioxidant and dopamine). Besides, extract and animal model(s) played an essential role in scientific phyto-research. In the last decade, the new designer drug started to be popular. Students have become an emerging part of new abusers of this medical plant.

## Discussion

This study conducts a visualized bibliometric analysis of the literature regarding *Catha edulis* from 1997 to 2020. During the past 24 years, *Catha edulis* publications from Ethiopia and Yemen increased due to the local prevailing major cash crops, legal use, and commercial trading. Consumed for socialization and leisure activities in East Africa and Arabian Peninsula, adults, adolescent students, and even pregnant women use *Catha edulis* daily; the estimated percentage of users in different countries and regions of East Africa and the southwestern Arabian Peninsula are extraordinarily high (Table 3) (Dimba et al., 2004; Al-Mugahed, 2008; Feyissa and Kelly, 2008; Odenwald and Al'Absi, 2017; Wondemagegn et al., 2017; Horn of Africa map, 2018). Gradually, *Catha edulis* has become prevalent in other parts of the world, such as Australia, Europe, North American countries, China, and Malaysia, owing to immigrant communities (Gebissa, 2010; Klein and Metaal, 2010), development of air transport (Kalix, 1992; Al-Hebshi and Skaug, 2005), and internet communication (Feyissa and Kelly, 2008). From the perspective of legislation, it is banned in the USA but is available and legal in some countries in Europe, including the UK, the Middle East, and Africa (Anderson and Carrier, 2009; Lemieux et al., 2015). So far, there are over 20 million *Catha edulis* abusers all over the world (Riyaz et al., 2014; Odenwald and Al'Absi, 2017), which causes emerging health concerns, such as psychiatric disorders, oral and cardiovascular disease, as well as corresponding research attention (Nichols et al., 2015). Theoretically, a legal restriction as an illicit drug would partly protect against the *Catha edulis* hazards in the USA (ACMD, 2013) and UK (Jones et al., 2014) compared to the producing regions or countries with the impact of cultural and traditional use (Gebissa, 2010). However, policies regarding the cultivation, transfer, trafficking, and use of *Catha edulis* are needed to be implemented. Public awareness should be launched to limit *Catha edulis* use and improve individual primary prevention activities.

**TABLE 3:** The estimated percentage of users in different countries/regions of East Africa and southwestern Arabian Peninsula.

Countries/regions	Percentage of C*atha edulis* users	
Saudi Arabia Jazan Region	43% males	11% females
Yemen	80–90% males	35–60% females 12–15% under 12 years
Djibouti	90%	
Ethiopia Harari (Hararghe) District	30–50%	
Somalia	18–55% males	10–25% females
Kenya	36.8%	
Kenya Coast & North Eastern District	88%	
Uganda	32%	

For Ethiopia, the USA, Saudi Arabia, and Yemen, as leading countries of the research output, the reasons could be possibly different. Except for the large abuse population in these countries, the USA, usually offering abundant research budgets to support high-level research activities, acted as the main driving force with a high academic reputation in scientific research and characteristic H-index value, while the legal cultivation, trade, and consumption of *Catha edulis* in Ethiopia and Yemen facilitate scientific research productivity (Gebissa, 2010).

The productive countries’ cooperation and research output are positive, especially among Ethiopia, the USA, and Saudi Arabia (Bozeman and Corley, 2004; Wagner, 2005; Huamani, 2015). Among these countries, Ethiopia and Saudi Arabia, also called the *Catha edulis* belt countries as main areas of commercial cultivation and consumption, could innately offer more information and collaborative opportunities, and the USA could be regarded as a financial grants and research collaboration provider to achieve higher productivity (Adams et al., 2014). With the prevalence of *Catha edulis* use, global cooperation should be promoted to improve research quality, and research outcomes should help recognize the necessity of legal restrictions and control of addiction at national levels (Douglas et al., 2011; Rahim et al., 2012).

The indicators, including the number of publications, citation per article, and H-index by a country, can be regarded as the quality of research activity in a specific field (Joshi, 2014; Luo et al., 2015). Our results show that the average citation per article on *Catha edulis* was 15.52, which was steadily increasing compared with the previous bibliometric results (Zyoud, 2015). Such vital information resources indicated that more attention was paid to the research related to *Catha edulis*. Though the average citations per article on tobacco and cannabis (25.27 and 27.23, retrieved by the WoS) were much higher than on *Catha edulis*, the citation growth on tobacco or cannabis were correspondingly declined compared to *Catha edulis* during the same period. This indicated that the amount of research focus on these two uncontrolled or divergent-legalization substances was steady, however, the yields of research attention poured into *Catha edulis* grew faster (Clermont et al., 2021).

Generally, the most cited articles are published in influential journals with high SJR or IF (Falagas et al., 2008; Kulkarni et al., 2009; Santa et al., 2010; de Granda-Orive et al., 2013). Our results show that more than half of the highly cited articles on *Catha edulis* focused on the adverse effects, and others related to mental health and harms to users and society were published in journals with IF 2020 < 10 and SJR 2020 < 4. Moreover, these top 10 highly cited articles were all published during 1999–2011. To some degree, this could be interpreted as these research performances gaining increasing attention in these years, which might help develop effective policies to gain public attention and prohibition measurements.

According to highly cited publications and keywords co-occurrence network, the active research area, including “prevalence”, “risk factor”, “chemical composition”, “toxicology”, and “pharmacology” over the past 24 years, are scoping and critical. According to these keywords, important research was retrieved. Similarly to amphetamine, a powerful psychotomimetic stimulant, *Catha edulis* produces various mental distress and psychotic symptoms such as irritability, insomnia, depression, reduced appetite, strange experiences, and hallucinations (Lemieux et al., 2015; Widmann, 2017; Abbay et al., 2018; Ongeri et al., 2019). The prolonged anorexia leads to low body weight in *Catha edulis* chewers and low birth weight of newborns in maternal users (Tesfay et al., 2019). The main chemical compounds of *Catha edulis*, cathinone, cathine, and norephedrine, account for all the psychostimulatory effects (Szendri, 1980). Cathinone, the natural amphetamine-like composition in *Catha edulis* with the highest levels in stems and young leaves, was proved to play a major role in this euphorising plant (Kalix, 1996; Alsanosy et al., 2020; Dhabbah, 2020). Some studies identified mechanisms of cathinone on the central nervous system in changing presynaptic striatal dopamine system and interfering with pituitary cell integrity in vervet monkeys (Nyongesa et al., 2014; Nyongesa et al., 2015), altering levels of dopamine and its metabolites and accelerating oxidative stress in limbic areas of swiss albino mice (Sathi et al., 2014; Safhi et al., 2018), inhibiting monoamine (dopamine, norepinephrine, etc) reuptake in human nerve cells (Bredholt et al., 2013), inducing striatal c-fos expression in Siberian hamster (Jones et al., 2014), and other complex mechanisms of psychosis caused by *Catha edulis* use (Odenwald et al., 2005; Bredholt et al., 2013). The network, combined with the co-occurring keywords, demonstrated the progress and correlation of the original research of *Catha edulis* at a global level *via* bibliometric mapping.

Besides, the highly cited article showed that researchers were interested in the potential genotoxic effects of *Catha edulis* by micronucleus assay with exfoliated cells in humans. In light of the pronounced increase in micronucleated buccal mucosa cells of volunteers who chewed *Catha edulis* regularly, it suggests that *Catha edulis* chewing may cause genetic damage and further lead to cancer in the oral cavity or other parts of the upper digestive tract (Kassie et al., 2001). About 50% of *Catha edulis* chewers develop keratosis of buccal mucosa, a pre-cancerous lesion, and 2–12% of individuals with such lesions develop oral cancer (Ahmed et al., 2011). On the other side, *Catha edulis* extracts have been shown to induce oral fibroblasts and keratinocytes apoptosis and arrest in G1-phase *in vitro*, which further adds to speculations about anti-carcinogenicity of *Catha edulis* (Al-Maweri et al., 2018). Moreover, *Catha edulis* has been implicated in causing other symptoms such as periodontitis, caries, gastritis, hypertension, and acute myocardial infarction (Al-Hebshi and Skaug, 2005). Other studies also proved that genetic factors had an important role to greatly deepen the understanding of toxicity. In all, such research focused on the complex adverse effects to health over the past years, however, these may provide potential targets for treatment in the future.

The co-cited clusters include “s-(-)-cathinone” and “norephedrine” from 2003 to 2016. S-(-)-cathinone, one of the enantiomers of cathinone, is more psychoactive than its R antipode and is detected only in *Catha edulis* (Alsanosy et al., 2020). Unstable cathinone in *Catha edulis* would mainly degrade to cathine and norephedrine within 48 h after harvest (Alsenedi and Morrison, 2018). Research revealed that norephedrine extracted from *Catha edulis* could induce T-lymphocyte proliferation in Swiss albino mice, which may result in liver and kidney injury and immune-stimulation (Ketema et al., 2015). Cathine and norephedrine can directly affect mammalian sperm function, such as accelerating capacitation, inhibiting spontaneous acrosome loss, and enhancing natural fertility at appropriate doses (Adeoya-Osiguwa et al., 2005). Furthermore, other constituents of *Catha edulis* such as tannic acid may be mutagenic and carcinogenic in human buccal mucosa cells, which might account for combining effects as well as other chemical compounds like flavonoid and polyphenolics (Kassie et al., 2001). Generally, this bibliometric result confirmed that more research was performed to investigate the main components of *Catha edulis*, like cathine and norephedrine, and quite a few noticed others, including tannic acid, flavonoid, and polyphenolics.

According to the co-cited timeline of *Catha edulis* research evolution, cortisol (#5) has been a focus for a long time (Liu et al., 2016). Interestingly, the retrieved studies indicated contradictory results that cortisol was reduced in male olive baboons with oral administration of *Catha edulis* oil to regulate hormones (Mwenda et al., 2006); by contrast, dose-dependent increase of cortisol in male SD rats and male NZW rabbits was associated with *Catha edulis* extract treatment (Nyongesa et al., 2008; Mohammed and Engidawork, 2011). However, the elevation of cortisol in humans was more obvious among chewers in the early evening than non-chewers in a cross-sectional study (Al’Absi et al., 2013). Additionally, Catha edulis-induced high serotonin levels in the human brain were associated with decreased testosterone, which can inhibit the release of cortisol (Muniyappa et al., 2010; Montoya et al., 2012; Lovallo, 2013; Lovallo et al., 2015). All the studies mentioned above on *Catha edulis*-induced cortisol changes are inconclusive, which may be due to differences in chemical components between the original plant and its extracts, or different administration methods, frequencies, and doses, or interaction between different drugs abused by addicts, or stress induced by its use (Al’Absi et al., 2013). Based on these, further research was recommended on cortisol regulation, like *Catha edulis* caused sympathetic excitation (Alshagga et al., 2016).

Among the keywords with the strongest burst citation, the “designer drug” burst since 2012 indicates another option of synthetic cathinones compared with traditional *Catha edulis* (Karila et al., 2015; Wang et al., 2020). Compared with mild stimulants used for better concentration and performance during trading, farming, socialization, and leisure activities, heavy use and/or concomitant abuse of substances (poly-drug use behavior, such as *Catha edulis* and a designer drug) due to stronger psychostimulatory desire is rapidly growing (Ali, 2018). Except for *Catha edulis*, the natural structural basis of emerging designer drugs, synthetic cathinones, as a preferred constitution and/or replacement, became increasingly abused (Anderson and Carrier, 2009; Risca et al., 2020). In face of such overwhelming social problems induced by a complex combination of original plant and structure-modified analogs, the research frontiers would be transferred to pharmacological and toxicological effects and abuse risks of synthetic cathinones such as methcathinone (MC), mephedrone (MEPH), 4-methylmethcathinone (4-MMC), 3-fluoromethcathinone (3-FMC), and methylenedioxypyrovalerone (MDPV) (Carlsson et al., 2018; Risca et al., 2020), or polydrug abuse.

Considering the keywords shared by Figures 4–6, researchers have been exploring the therapeutic value of *Catha edulis* for decades. In the past, *Catha edulis* was traditionally perceived to treat headaches, common cold, and respiratory diseases (Al-Hebshi and Skaug, 2005). In recent years, many medicinal values of Catha edulis have been discovered gradually. Callus of Catha edulis have “HIV-1” reverse transcriptase inhibition effects and exhibit high antibacterial properties against both gram-positive and gram-negative bacteria compared to plant leaves (Kumari et al., 2015). Besides, the “medical plant” induces apoptosis in human breast cancer MDA-MB-231 cells *via* sustainable activation of C-Jun NH2-terminal kinase (JNK) and MAPK, and mitochondrial-mediated apoptosis pathway, which suggests that *Catha edulis* has substantial potential as a source of anticancer agents (Bredholt et al., 2009; Lu et al., 2017). Accordingly, the different effects of *Catha edulis* on different types of cancer cells might be a future research direction for medicinal potentials.

Moreover, the bibliometric results noticed other properties of Catha edulis. High dose *Catha edulis* extract, cathinone, blocked the body weight gain of male mice on an obesity genic diet through upregulating lipolytic genes activity in white adipose tissue (Alshagga et al., 2020). Cathine in *Catha edulis* acted as an effective weight loss agent for adjunct treatment of obesity with significant weight loss in overweight and obese patients (Hauner et al., 2017). Poly (vinyl alcohol) hydrogels by cellulose nanofibers (CNFs) originated from *Catha edulis* have been prepared in the field of tissue engineering applications. The hydrogels, with favorable mechanical, thermal properties, biodegradation nature, and antimicrobial activities against pathogenic bacteria, are suitable for neural substitutes (Abdel Bary and Harmal, 2019a; Abdel Bary and Harmal, 2019b). More similar scientific studies could be future focus areas for more comprehensive medicinal plant research.

## Conclusion

We made a systematic bibliometric assessment of the literature on *Catha edulis* from 1997 to 2020 and constructed a series of visualized graphs to elucidate the progress and emerging trends of the research. As an increasingly popular natural stimulant, the research trend was to explore not only phytochemical constituents and biological activities, but also toxicological and pharmacological effects and abuse threats, as well as the clinical therapeutic potential. This study is essential to raise public awareness of limited use and primary prevention activities of *Catha edulis*; furthermore, multidisciplinary efforts will still be needed to understand the further mechanisms of carcinogenic or antitumor effects, pathogenicity, and other medical values in the future. Getajiu and Krikoriaand, 1983, Kamdem et al., 2019, Miro et al., 2009, Regunath et al., 2012, Zyoud et al., 2017.

## Limitations

There were inevitable limitations to our study. First, the science literature database (WoS) keeps constantly publishing from time to time, and the time lag between the publication and the retrieval of publications might affect the time-sensitivity of the research. Second, in order to use the co-occurrence and co-citation analysis methods by CiteSpace, only English studies were analyzed due to the incompatibility of multiple languages in the software. Last but not least, the full taxonomic name “*Catha edulis* (Vahl) Endl.” was seldom used in previous publications; instead of “*Catha edulis*”, it is not sufficient for a scientific name but suitable for bibliometric research.

## Data Availability

The raw data supporting the conclusion of this article will be made available by the authors, without undue reservation.
